# Bacteria Belonging to *Pseudomonas typographi* sp. nov. from the Bark Beetle *Ips typographus* Have Genomic Potential to Aid in the Host Ecology

**DOI:** 10.3390/insects11090593

**Published:** 2020-09-03

**Authors:** Ezequiel Peral-Aranega, Zaki Saati-Santamaría, Miroslav Kolařik, Raúl Rivas, Paula García-Fraile

**Affiliations:** 1 Microbiology and Genetics Department, University of Salamanca, 37007 Salamanca, Spain; nier.ezep@usal.es (E.P.-A.); zakisaati@usal.es (Z.S.-S.); raulrg@usal.es (R.R.); 2 Spanish-Portuguese Institute for Agricultural Research (CIALE), 37185 Salamanca, Spain; 3 Department of Botany, Faculty of Science, Charles University, Benátská 2, 128 01 Prague, Czech Republic; miroslavkolarik@seznam.cz; 4 Laboratory of Fungal Genetics and Metabolism, Institute of Microbiology of the Academy of Sciences of the Czech Republic, 142 20 Prague, Czech Republic; 5 Associated Research Unit of Plant-Microorganism Interaction, University of Salamanca-IRNASA-CSIC, 37008 Salamanca, Spain

**Keywords:** *Ips typographus*, bark beetle, bacteriome, *Pseudomonas*, beneficial

## Abstract

**Simple Summary:**

European Bark Beetle (*Ips typographus)* is a pest that affects dead and weakened spruce trees. Under certain environmental conditions, it has massive outbreaks, resulting in attacks of healthy trees, becoming a forest pest. It has been proposed that the bark beetle’s microbiome plays a key role in the insect’s ecology, providing nutrients, inhibiting pathogens, and degrading tree defense compounds, among other probable traits. During a study of bacterial associates from *I. typographus*, we isolated three strains identified as *Pseudomonas* from different beetle life stages. In this work, we aimed to reveal the taxonomic status of these bacterial strains and to sequence and annotate their genomes to mine possible traits related to a role within the bark beetle holobiont. Our study indicates that these bacteria constitute a new species for which the name of *Pseudomonas typographi* sp. nov. is proposed. Moreover, their genome analysis suggests different metabolic pathways possibly related to the beetle’s ecology. Finally, in vitro tests conclude the capability of these bacteria to inhibit beetle’s fungal pathogens. Altogether, these results suggest that *P. typographi* aids *I. typographi* nutrition and resistance to fungal pathogens. These findings might be of interest in the development of integrated methods for pest control.

**Abstract:**

European Bark Beetle *Ips typographus* is a secondary pest that affects dead and weakened spruce trees (*Picea* genus). Under certain environmental conditions, it has massive outbreaks, resulting in the attacks of healthy trees, becoming a forest pest. It has been proposed that the bark beetle’s microbiome plays a key role in the insect’s ecology, providing nutrients, inhibiting pathogens, and degrading tree defense compounds, among other probable traits yet to be discovered. During a study of bacterial associates from *I. typographus*, we isolated three strains identified as *Pseudomonas* from different beetle life stages. A polyphasic taxonomical approach showed that they belong to a new species for which the name *Pseudomonas typographi* sp nov. is proposed. Genome sequences show their potential to hydrolyze wood compounds and synthesize several vitamins; screening for enzymes production was verified using PNP substrates. Assays in Petri dishes confirmed cellulose and xylan hydrolysis. Moreover, the genomes harbor genes encoding chitinases and gene clusters involved in the synthesis of secondary metabolites with antimicrobial potential. In vitro tests confirmed the capability of the three *P. typographi* strains to inhibit several *Ips* beetles’ pathogenic fungi. Altogether, these results suggest that *P. typographi* aids *I. typographi* nutrition and resistance to fungal pathogens.

## 1. Introduction

European bark beetle *I. typographus* is a secondary pest that affects mainly dead, stressed, and weakened spruce trees (*Picea* genus). Under certain environmental conditions, they have massive outbreaks, resulting in their attack of healthy trees, thus becoming a forest pest. It has been proposed that bark beetles’ microbiome plays a key role in the insect’s ecology with the provision of nutrients, inhibition of pathogens, degradation of tree defense compounds, among other probable traits yet to unveil [1,2].

The bark beetle *I. typographus* is a phloem-feeding species. Its main life cycle occurs inside the inner bark of their host. When environmental conditions and host availability are appropriate, male adults seek for a new host, usually recently tumbled or diseased trees. Once the host is found, pheromones are emitted as a signal call for other individuals to infest the tree. Adult bark beetles perforate the external bark to reach the inner bark, where their life cycle will be developed. Adults excavate galleries, and eggs will be laid. Once larvae are born, they excavate their own galleries, develop, and proceed through all the life stages (pupae, young adult, and adults). Thus, almost all their life cycle develops inside the inner bark, which is mainly composed of cellulose, hemicellulose, and lignin [1,3,4]. *Picea* trees execute a chemical defense when a beetle attack is detected; this defense is mainly mediated via terpenoid and phenolic compounds. It has been described that beetles must be on an outbreak (mass attack) to surpass a healthy tree’s defense and that the microbiota could assists the beetle to tolerate this tree’s chemical defense by degrading such aromatic compounds [5,6]. Both lignocellulosic hydrolysis and aromatic compound degradation via microbiota is theorized as a supplementary energy, nutrient, and/or essential biomolecules source for the beetle [1,4]. Some microbes associated with the beetle are able to synthesize a constellation of enzymes—e.g., cellulases, hemicellulases, oxidases, and peroxidases—which hydrolyze those complex molecules into sugars and low molecular weight nutrients, which can be assimilated by the microbes themselves or by the beetle [1,4].

Besides that, tree’s phloem, the main feed source for the beetle, has been described as a sugar-rich source but poor in phosphorus, nitrogen, and vitamins, among other relevant nutrients [7,8,9], and it has been suggested that the provision of these scarce substances could be a role of the beetle microbiota [1]. Phosphorus and nitrogen are essential nutrients for live development. In this scenario, phosphorus could be obtained from biomolecules produced by the tree, which are non-assimilable for the beetle, and nitrogen could be recycled from various sources or fixed from the environment by associated microbes [1,2]. Wood is deficient in B-vitamin and beetles cannot synthesize this essential vitamin, which could be synthesized by its microbiome [8].

On the other hand, some of the organisms that beetles encounter on and in their spruce host are pathogens, including various protists, nematodes, virus, bacteria, and fungi [10,11,12]. It has also been proposed that the microbiota could protect the beetles from these enemies, mainly by producing antimicrobial compounds [1,13].

Bark beetles mycobiota have been deeply studied; fungal endosymbionts have been identified, and the role of filamentous fungi and yeast role in beetle ecology has been thoroughly described [2,8,14]. Recently, it has been proposed that the beetle’s bacteriome could also be playing important roles for its host, such as providing nutrients, facilitating the access to the phloem, improving defense to pathogens, or degrading the tree’s aromatic compounds, among others [1,2,3,4,5,6,13,15].

It has already been described that the beetle’s microbiome is relatively rich in bacterial diversity, including a wide range of genera [1,2,15,16], and new bacterial species have been isolated from the interior of bark beetles, e.g., from *Ips* beetles: *Erwinia typographi* or *Pseudomonas bohemica* [13,17].

*Pseudomonas* is a bacterial genus that has been repeatedly described as part of the *Ips* microbiome [4,13,15,16]. The *Pseudomonas* genus is known as a cosmopolitan group with a wide metabolic machinery, which includes (but is not limited to) the production of antibiotic compounds, hydrolytic enzymes, siderophore molecules, vitamins, and aromatic compounds degradation enzymes [1,4,18,19].

The present research is a part of a wider study aiming to decode *I. typographus* bacteriome and its role in the beetle holobiont. Three *Pseudomonas* strains were isolated from *I. typographus* adults (strain CA3A) and larvae (strains C2L11 and C2L12B) and characterized; a preliminary analysis of their 16S rRNA gene sequence made us hypothesize that they could constitute a new species within the genus *Pseudomonas*. Moreover, their presence in different life stages of the beetle (larvae and adults) suggested that they could be playing an important role in the insect’s ecology. Thus, in this work, we aimed to reveal the taxonomic status of these bacterial strains and to sequence and annotate their genomes in order to mine possible traits related to a role within the bark beetle holobiont. According to a polyphasic taxonomic approach, they seem to constitute a new species within the genus, for which the name of *Pseudomonas typographi* sp. nov. is proposed and CA3A^T^ is designated as a type strain. Moreover, the genome sequences of these three isolates reveal several metabolic pathways that might be related to the beetle’s ecology. Finally, in vitro tests were done to these strains to evaluate some of the most relevant predicted metabolic capabilities of *P. typographi* strains, concluding a remarkable capability to aid *I. typographus* nutrition and resistance to fungal pathogens.

## 2. Materials and Methods

### 2.1. Bacterial Isolation

Logs of *Picea abies* were collected from a forest in Nižbor (49°59′42.4″ N, 13°56′49.7″ E), Czech Republic. After the bark was removed, pools composed by 10 *I. typographus* individuals of different stages (larvae, pupae, and adults) were recollected and surface sterilized for 1 min with HgCl_2_ (2%) and cleared with distilled water on aseptic conditions.

Afterwards, insects were crushed, and serial dilutions were made. Finally, solutions were inoculated at different concentrations (10^−3^ to 10^−6^) on tryptic soy agar (TSA, Sigma-Aldrich, Darmstadt, Germany).

Individual colonies were picked up until pure cultures were obtained. For identification purposes, isolates from adults were labeled CA and those from larval stage 2 were labeled C2L, which was followed by correlative numbers for each isolate. Pure cultures were stored in 20% glycerol and at −80 °C for long-term preservation.

### 2.2. DNA Extraction and Bacterial Identification

For bacterial strains identification, REDExtract-N-Amp™ Tissue PCR Kit Protocol (Sigma-Aldrich, Darmstadt, Germany) was used as indicated by the manufacturer to extract DNA. The 16S rRNA gen was amplified as described in Rivas et al. (2007) [20]. PCR products were visualized on a 1% agarose gel after electrophoresis (60 V for 120 min). Purification was done following the manufacturer’s protocol with GeneJET Gel Extraction and a DNA Cleanup Micro Kit (Thermo Scientific^TM^, Göteborg, Sweden). Purified PCR products were sequenced at Macrogen Ltd. (Madrid, Spain). The sequence fragments obtained were aligned using BioEdit [21], and the resulting sequences were compared against those of type strains on the EzBioCloud 16S rRNA database using the EzTaxon-e online server [22].

For genome sequencing, total genomic DNA was obtained with Quick-DNA^TM^ Fungal/Bacterial Miniprep Kit (Zymo Research, Orange, CA, USA) following the manufacturer’s instructions. A genome sequence was performed on an Illumina MiSeq platform with 2 × 250 bp paired end reads at Microbes NG Ltd. (Birmingham, UK). Sequences’ de novo assembling was done using SPAdes v3.13.0 [23]. Genome sequences were deposited at the NCBI’s GenBank database under the Bioproject PRJNA610756. BioSample and Genome Accessions are, respectively, SAMN14311041 and JAAOCA010000000 for strain CA3A^T^, SAMN14311042 and JAAOCB010000000 for strain C2L11, and SAMN14311043 and JAAOCC010000000 for strain C2L12B.

### 2.3. Phylogenetic Analyses

The phylogenetic analyses of the 16S rRNA gene sequence and the concatenated sequences of the *gyrB*, *rpoB*, and *rpoD* housekeeping genes of strains CA3A^T^, C2L11, and C2L12B were done using gene sequences obtained from the genome [13].

Sequences of housekeeping genes of the closely related type strains of the genus *Pseudomonas* were selected within the most similar ones shown on the BLASTn alignment tool (Web Suite) using default settings and searching only among the type material on the Nucleotide collection nr/nt database. Analysis and tree construction were performed on MEGA X software [24], which were based on the ClustalW all nucleotides alignment [25,26]. The initial tree for the heuristic search was obtained automatically by applying Neighbor-Join and BioNJ algorithms to a matrix of pairwise distances estimated using the Maximum Composite Likelihood (MCL) and the phylogenetic trees were generated following the Maximum Likelihood method and Tamura-Nei model [27] analyses. The average nucleotide identity based on BLAST (ANIb) values between the genome sequence of strains CA3A^T^, C2L11, and C2L12B and the genome sequences of the type strains of the closest related species were estimated by using PYANI (Python module for average nucleotide identity analyses) software (v0.2.1) [28]. A clustered heatmap was constructed with R-project by calling heatmap.2 from the gplots package [29].

The genome-to-genome distance calculator (GGDC) tool offered by DSMZ [30] was used: (i) between the three different strains and (ii) with the closest type strains according to the phylogenetic and ANIb analyses. This technic is a state-of-the-art in silico method for inferring whole-genome distances, which are well able to mimic DDH (DNA–DNA hybridization), so-called digital DDH or dDDH; Formula 2 (identities/ high-scoring segment pairs (HSP) length), which is recommended for distance calculation by DSMZ, was used in this study.

### 2.4. Genomic Analyses

Gen-calling and annotation of the draft genomes were performed on RAST (Rapid Annotations using Subsystems Technology) (v2.0) [31]. KEGG Orthology (KO)-based annotation was performed with KofamKOALA [32]. Analysis for the in silico potential role in the beetle’s life cycle of these strains was studied through metabolic pathways analyses in “The SEED-viewer” [33] and KofamKOALA. AntiSMASH (v5.1.0) [34] was used as a specific complement for the annotation of secondary metabolite biosynthetic gene clusters (BGCs). Carbohydrate-active enzymes (CAZYmes) were predicted on a DBCAN2 meta server using HMMER, DIAMOND and HotPep algorithms and, as developers recommend [35], only those CAZYmes detected by at least two tools were taken into consideration.

The G + C content in mol% of DNA was calculated from the genome sequences with RAST (v2.0).

### 2.5. Lignocellulose-Related Activity

To evaluate the capacity of strains CA3A^T^, C2L11, and C212B to produce wood compounds hydrolytic enzymes, p-nitrophenyl (pNP)-bounded substrates were used (see Table 1). First, 0.2% concentrated substrates were prepared in 0.2 M phosphate buffer (pH 7). Then, 80 µL of this substrate was mixed up with 80 µL of a bacterial suspension at McFarland 7 standard concentration obtained from cell cultures grown in Petri dishes with TSA medium for 7 days at 28 °C. Four replicas per strain and substrate were prepared. Each bacterial solution–substrate mix was placed in different wells of a multi-well plate and left for incubation for 48 h at 28 °C. After incubation, the plate was revealed with a 4% sodium carbonate solution, adding 135 µL per well. A positive reaction was evidenced by the appearance of a yellow color within a few seconds.

In vitro hydrolysis of bark and wood polysaccharides (cellulose, xylan, starch, and pectin) was tested in Petri dishes with the corresponding substrate added to a TSA medium as previously reported [4,36,37,38,39]. Carboxymethyl cellulose (CMC) (Sigma-Aldrich, Darmstadt, Germany) at 0.2% was added into the medium as source of non-soluble cellulose for cellulase activity assessment. Beechwood xylan (Sigma-Aldrich, Darmstadt, Germany), potato starch (Sigma-Aldrich, Darmstadt, Germany), and citrus peel pectin (Sigma-Aldrich, Darmstadt, Germany) at 1% concentration were respectively added to the media for xylanase, amylase, and pectinase activity assessment. Drops of 5 µL of a bacterial suspension at a McFarland 7 standard were inoculated over the plate’s surface. After incubating for a week at 28 °C, colonies were carefully washed out with sterile water. CMC and xylan plates were stained using 0.1% Red Congo (Panreac Química SLU, Barcelona, Spain) solution for 30 min, while starch and pectin plates were revealed in the same way but using a Lugol’s solution (Panreac Química SLU, Barcelona, Spain) as a dye. Three 15-min washes of NaCl (1 M) solution were performed for dye excess removing.

### 2.6. Antimicrobial Potential

The inhibition of *Lecanicillium muscarium* CCF 3297, *Beauveria brongniartii* CCF 1547, *Metarhizium anisopliae* CCF 0966, *Beauveria bassiana* CCF 5554, *Lecanicillium muscarium* CCF 6041, *Isaria farinosa* CCF 4808, *Beauveria bassiana* CCF 4422, and *Isaria fumosorosea* CCF 4401 fungi was tested. These fungal strains were selected as they are potential pathogenic fungi for *Ips* beetles [40,41,42]. Inhibition tests were done by streaking 3 lines of strains CA3A^T^, C2L11, and C2L12B on TSA medium plates. Plates were incubated for a week (28 °C) to allow bacterial growth and antimicrobials compounds production and diffusion in the agar. After the incubation time, fungal plugs of mycelium from each fungus were placed over these plates at about 1.5–2 cm distance from the bacteria and incubated for a week at 28 °C. Fungal growth without the presence of the bacteria was also tested in same conditions (negative control). After the incubation time, fungal growth was checked and compared with that of the control plates.

Since siderophores are iron scavenger molecules, which have been related to the inhibition of other microbes, siderophore production was evaluated on modified M9-CAS-agar medium plates as described by Jiménez-Gómez et al. [43]. Plates inoculated with 5 µL bacterial suspensions prepared as indicated in Section 2.5 were incubated at 28 °C for a week. An orange halo around the colonies was considered as a positive result.

### 2.7. Bacterial Characterization

Gram staining of strains CA3A^T^, C2L11, and C2L12B was carried out following the protocol described by Doetsch (1981) [44]. The type of flagellation was determined by electron microscopy as previously described by García-Fraile et al. (2015) [45].

A minimal medium broth supplemented with 0–7% (*w*/*v*) NaCl was used to assay salt tolerance in CA3A^T^, C2L11, and C2L12B. The same base medium with an adjusted final pH in the range of 4 to 10 was used for studying the growth capability of the strain at different pHs; in both cases, the cultures were incubated in a shaker (180 r.p.m.) at 28 °C for up to 1 week. In addition, cells cultured on the same minimal medium were grown at 4, 12, 28, and 37 °C to determine the temperature range for growth. In all cases, the presence of growth was checked for 1 week.

For the catalase test, bacterial cells grown for three days at 28 °C in a minimal medium (K_2_HPO_4_ 0.3%, KH_2_PO_4_ 0.3%, MgSO_4_ + 7H_2_O 0.15%, CaCl_2_ + 2H_2_O 0.05%, NaCl 0.1%, NH_4_NO_3_ 0.1%, Mannitol 10% and 2% agar) were collected, and drops of 30% H_2_O_2_ were added over them to detect the formation of bubbles after 5 min, indicating a positive result. The oxidase test was performed following the protocol described by Kovacs (1956) [46].

Finally, the phenotypic characterization of strains CA3A^T^, C2L11, and C2L12B was done using API 20 NE and 50 CH tests (bioMérieux^®^, Marcy-l’Etoile, France) as manufacturer instructions indicated. API 50 CH tests were done with a minimum medium without carbon source and bromocresol purple as a pH indicator.

## 3. Results

### 3.1. Bacterial Identification

As a part of a broader work aiming to study bacteria associated to bark beetles (unpublished), we isolated strains CA3A^T^, C2L11, and C2L12B, which, based on the nearly complete (approximately 1400 pb) 16S rRNA gene sequence identification, were identified as *Pseudomonas* but showed a low similarity with their closest related species *P. congelans* DSM 14939T (97.4%), *P. meliae* Ogimi 2^T^ (97.2%), and *P. silesiensis* A3T (97.2%), indicating that they might conform a new *Pseudomonas* species.

After genome sequences were obtained, complete 16S rRNA and housekeeping *gyrB*, *rpoB¸* and *rpoD* sequences were used for further comparison with the closest *Pseudomonas* type strains.

**Figure 1:**
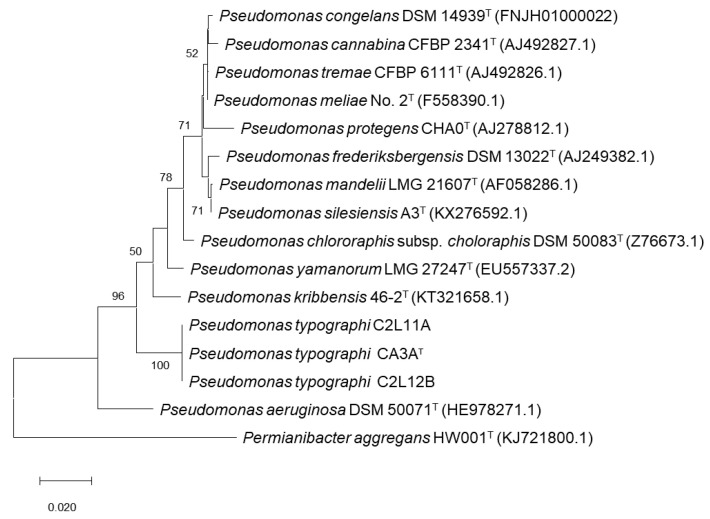
Maximum-likelihood phylogenetic tree based on complete 16S rRNA gene sequences of all *Pseudomonas* species closely related to *P. typographi* CA3A^T^, C2L11, and C2L12B, *P. aeruginosa* DSM50071^T^, and *P. aggregans* HW001^T^. Bootstrap values (expressed as percentages of 1000 replications) are shown at the branching points; values below 50 were discarded. Scale bar = 2 nucleotide (nt) substitutions per 100 nt. Accession numbers of the sequences are indicated in parentheses.

**Figure 2:**
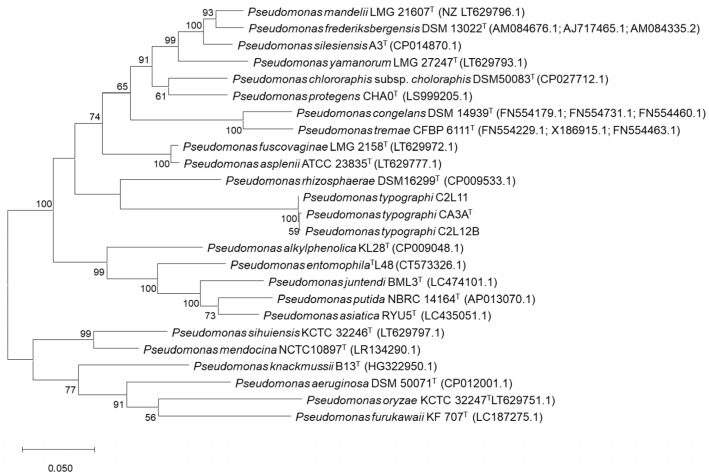
Maximum-likelihood phylogenetic tree based on concatenated partial *gyrB*, *rpoB*, and *rpoD* gene sequences of strains *P. typographi* CA3A^T^, C2L11, and C2L12B and closely related species of the genus *Pseudomonas*. Bootstrap values (expressed as percentages of 1000 replications) are shown at the branching points; values below 50 were discarded. Scale bar = 5 nucleotide (nt) substitutions per 100 nt. GenBank accession numbers of the sequences are indicated in parentheses.

An ML (Maximum Likelihood) phylogenetic tree including all related species within the genus *Pseudomonas* that showed an identity over 97% on the 16S rRNA gene sequence was performed. *P. aeruginosa* DSM50071^T^ was included as the type species of the genus and *Permianibacter aggregans* HW001^T^ was included as an outgroup (Figure 1). This analysis showed that strains CA3A^T^, C2L11, and C2L12B cluster together and aside of every closest *Pseudomonas*-type strain included in the analysis. As described by Ramírez-Bahena et al. (2014) [47], analysis based only on the 16S rRNA sequence is very limited to discriminate species within the genus *Pseudomonas* at the inter-species level. In consequence, a Multi-Locus Sequence Analysis (MLSA) based on the three housekeeping genes *gyrB*, *rpoB*, and *rpoD* was performed to locate these strains within the genus *Pseudomonas* [47,48,49,50,51,52,53,54]. ML analysis of the concatenated housekeeping *gyrB*, *rpoB,* and *rpoD* genes sequences (Figure 2) showed that strains CA3A^T^, C2L11, and C2L12B cluster together in an independent branch of any other type strain of *Pseudomonas* genus included in the analysis, forming a clade with *P. rhizosphaerae* DSM16299^T^, which appears in a very distant branch within this clade, suggesting the assignment of the strains of this study to a new species within the genus *Pseudomonas*.

**Figure 3:**
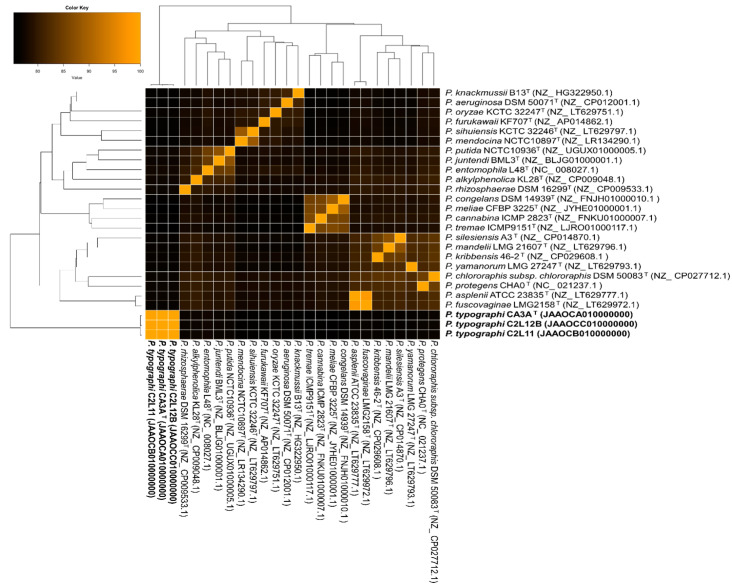
Average nucleotide identity based on BLAST (ANIb) heatmap, clustered heatmap constructed with gplots package based on ANIb values obtained with the PYANI platform for all 27 genomes. GenBank accession numbers of the sequences are indicated in parentheses.

To compare the genome sequences of the three strains and the genomes of the closest related species of the genus, ANIb values shared among them were calculated, and a clustered heatmap was performed (Figure 3). The results showed that the range of similarity of these three strains to the closest *Pseudomonas*-type strains is between 75.3% and 77.6%. Most of the related species were *P. entomophila* (77.5%), *P. alkylphenolica* (77.3%), and *P. rhizosphaerae* (77.2%).

An in silico DNA–DNA hybridization (DDH) using the genome-to-genome distance calculator (GGDC) calculator was done to assess (i) if all three strains were, within them, the same *Pseudomonas* species and (ii) their relationship to the closest type strains selected of previous phylogenetic analysis. Results showed that the CA3A^T^–C2L11 dDDH value is 99.6%, the CA3A^T^–C2L12B value is 97.7%, and the C2L11–C2L12B value is 97.7% (identities/HSP length formula), which means that the probability, calculated with a logistic regression, of belonging to the same species is 97.7% or above. Furthermore, all three genomes showed that they are distant enough to be considered a different species to their closest relatives (Table 2). This analysis also confirmed that *P. entomophila*, *P. alkylphenolica*, and *P. rhizosphaerae* are the closest type strains to *P. typographi* CA3A^T^, C2L11, and C2L12B (Table 2).

### 3.2. Genome Analysis

A genome general description based on RAST analysis is shown in Table 3. The total genome size of *P. typographi* CA3A^T^, C2L11, and C2L12B is approximately 5.9–6.1 million pb and the G + C content is 62.0–62.2. The number of contigs obtained after the assembling process of the genome sequences was 229 for CA3A^T^, 158 for C2L11, and 174 for C2L12B. The gene calling predicted 5800–6000 coding sequences (CDSs) and 60–64 RNAs sequences for these genomes. Annotated features were classified into 364 subsystems in strains CA3A^T^ and C2L11B and in 362 for C2L11, understanding a subsystem as a collection of functional roles that are associated to each other in a wider system; approximately 26–27 of the predicted CDSs belong to a subsystem and from these, about 97 are non-hypothetical proteins. Regarding the remaining 74 CDSs, around 41–43 are hypothetical proteins.

According to the KofamKOALA annotations, all three strains isolated in this study were predicted to contain all necessary genes of the metabolic pathway to reduce adenyl sulfate (APS) (EC 2.7.7.4) to 3′-phosphoadenylyl sulfate (PAPS) (EC 2.7.1.25), this to sulfite (EC 1.8.4.8) and finally, to assimilable sulfide (EC 1.8.1.2).

Moreover, the genomes of *P. typographi* CA3A^T^*,* C2L11, and C2L12B have genes annotated as encoding proteins involved in the biosynthesis of thiamine (vit. B1), riboflavin (vit. B2), pyridoxine (vit. B6), nicotinate (vit. B3), biotine (vit. B8), and folate (vit. B9).

Regarding aromatic compounds degradation, all three strains have predicted metabolic pathways related to *p*-hydroxybenzoic acid (PHBA) degradation. In addition, a gene encoding for the salicylate hydroxylase enzyme (EC 1.14.13.1) was annotated, which catalyzes the conversion of salicylate into catechol.

Secretion systems type II and Sec-SRP were predicted for all three strains. Moreover, for strains C2L11 and C2L12B, gene encoding secretion systems type I and IV were also annotated. These systems were not predicted in the genome sequence of strain CA3A^T^.

The AntiSMASH program predicted the same five BGCs for all three strains: (i) a bacteriocine cluster that does not resemble any biosynthetic gene cluster (BGC) in the AntiSMASH database but, according to BLASTp, the core gene shows 64.5% similarity to a DUF692 family protein of *Shinella kummerowiae,* (ii) a Non-Ribosomal Peptide Synthetase-Like (NRPS-Like) cluster, in which 40% of genes show similarity to L-2-amino-4-methoxy-trans-3-butenoic acid (AMB) from *P. aeruginosa* PAO1, (iii) a NRPS cluster in which 34% of genes show similarity to a crochelin A biosynthetic gene cluster from *Azotobacter chroococcum* NCIMB 8003, (iv) a NAGGN cluster that does not resemble any known cluster in antiSMASH database but, according to the BLASTp comparison, its three core biosynthetic genes are similar to an N-acetylglutaminylglutamine synthetase (88.4% similarity to *Pseudomonas massiliensis*), an N-acetylglutaminylglutamine amidotransferase (88.3% similarity to *Pseudomonas fluorescens*), and an osmoprotectant N-acetylglutaminylglutamine amide (NAGGN) system M42 family peptidase (87.8% similarity to *Pseudomonas fluorescens*), respectively, and (v) a cluster related to the biosynthesis of a carotenoid molecule, in which 100% of the genes show similarity to that of *Enterobacteriaceae bacterium* DC413.

The potential of the three strains to hydrolyze plant polysaccharides and the fungal cell wall was analyzed through the annotation of Carbohydrate-Active Enzymes (CAZymes). The complete list of annotated CAZymes is detailed in Table 4. *P. typographi* strains CA3A^T^, C2L11, and C2L12B genome sequences annotated 16 GH families; among these, the GH 3 and GH 39 families include β-glucosidases (EC 3.2.1.21), β-xylosidases (EC 3.2.1.37), and α-l-arabinofuranosidase (EC 3.2.1.55). The GH 3 family also include exo-1,3/1,4-β-glucanase (EC 3.2.1) and exo-xyloglucanase (EC 3.2.1.155). The GH 10 and 13 families include α-amylase (EC 3.2.1.1) and α-glucosidase (EC 3.2.1.20), while the GH 15 family includes glucoamylase (EC 3.2.1.3). GH 19 and 23 include chitinases (EC 3.2.1.14) and the GH family 94 includes cellobiose phosphorylase (EC 2.4.1.20), cellodextrin phosphorylase (EC 2.4.1.49), and cellobionic acid phosphorylase (EC 2.4.1.321). In addition, the three genomes were predicted to harbor other CAZymes such as auxiliary activities (AA), carbohydrate esterases (CE), and polysaccharide lyases (PL). The AA 3 family includes cellobiose dehydrogenase (EC 1.1.99.18), the CE 4 family includes acetyl xylan esterase (EC 3.1.1.72), and PL 4 and 26 include rhamnogalacturonan endolyase (EC 4.2.2.23) and rhamnogalacturonan exolyase (EC 4.2.2.24), respectively.

### 3.3. In Vitro Assays of Catalytic Reactions

In vitro assays of p-nitrophenyl (pNP) substrates for strains CA3A^T^, C2L11, and C2L12B were used to test a selection of five different enzymatic reactions related to wood polysaccharide hydrolyzation. All three strains showed a weak-positive result for the degradation of α-glucosidase, β-glucosidase, α-xylosidase, β-xylosidase, and cellobiohydrolase.

Petri dishes containing TSA medium that incorporated CMC, xylan, starch, or pectin were inoculated with solutions of *P. typographi* CA3A^T^, C2L11, and C2L12B strains at McFarland 7 concentration. The presence of halos on all four different substrates confirmed that all three strains could hydrolyze these substrates.

### 3.4. Antibiosis Potential

In relation to the antimicrobial potential, the capability of the strains to synthesize siderophores and to inhibit fungal entomopathogens was tested. In vitro analyses for the detection of the predicted synthesis of siderophore molecules were performed on modified M9-CAS-agar medium plates. After inoculation, halos were appreciated within the first 24 h for the three strains.

**Figure 4:**
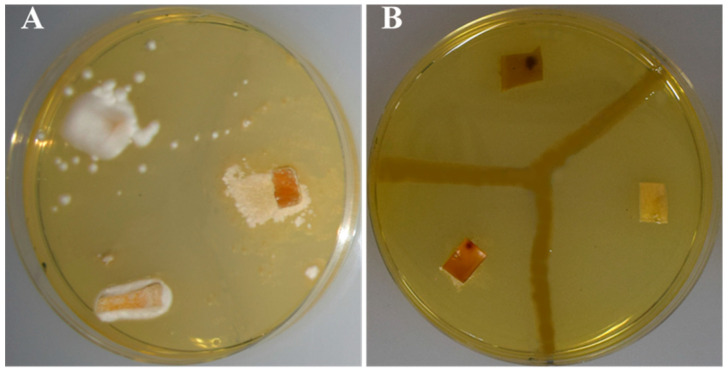
Antibiosis assays against bark beetle fungal pathogens. (**A**) Control plate with fungal plugs and no bacteria. (**B**) Plate streaked with strain C2L11. In (**A**,**B**), superior left plug corresponds to *Beauveria bassiana* CCF5554, right plug is *Lecanicillium muscarium* CCF6041, and inferior left is *Isaria farinosa* CCF4808.

Antibiosis assays against bark beetle fungal pathogens showed that all three strains of this study are capable of inhibiting fungal pathogens. Nonetheless, whereas strain C2L11 showed a total growth inhibition halo, strains CA3A^T^ and C2L12B just inhibited the total growth of some of the fungal strains, whereas for other pathogenic fungal strains, just aerial mycelium was inhibited; therefore, it was considered as a partial inhibition (Figure 4 and Table 5).

### 3.5. Other Phenotypic Traits of P. typographi

*P. typographi* strains CA3A^T^, C2L11, and C2L12B showed an optimum growth after 48 h incubation at 28 °C on TSA medium, exhibiting the typical iridescent morphology of this genus, although growth was confirmed in the temperature range of 4 to 37 °C. Regarding pH, growth was observed in the range of 6 to 10, but not at pH 4, showing the optimum growth at pH 8. All three strains were able to grow with 0 to 7% NaCl concentration in minimal medium, showing optimal growth with 1% NaCl.

**Figure 5:**
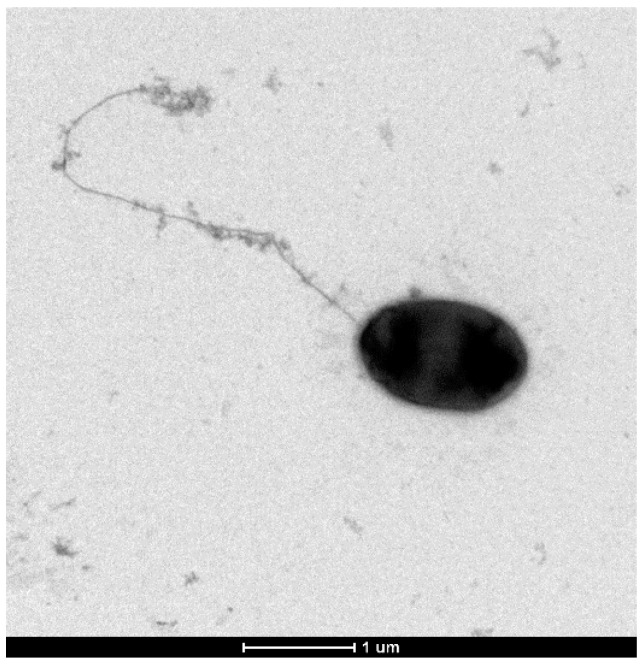
Transmission electron microscopy (TEM) micrograph showing the shape, size, and flagellum of *P. typographi* CA3A^T^.

Gram staining showed Gram-negative bacteria and electronic microscopy revealed rod-shaped cells with a polar flagellum (Figure 5).

After the addition of 30 hydrogen peroxide over the cells of the three strains, the presence of bubbles confirmed catalase activity and blue-stain presence after Kovac’s reactive addition confirmed oxidase activity.

Other phenotypic features of *P. typographi* sp. nov. are detailed in the prologue, and those differences with the closely related species are detailed in Table 6.

## 4. Discussion

Several different recent researchers have suggested important roles of the bacterial associates in the bark beetle holobiont, but it is a field that is yet to be described in depth. Some of these bacteria are indeed new bacterial species; e.g., *P. bohemica* was firstly isolated from *I. acuminatus* and its type strain was described as capable of inhibiting several microbial strains [13]. In addition, cellulolytic activity was described for the type strain of *P. coleopterorum,* first isolated from *Hylesinus fraxini* [54]. Bark beetles cannot digest these compounds, which otherwise would be a potential source of carbon [4,15,54].

In this study, strain CA3A^T^ was isolated from an adult bark beetle from the species *I. typographus;* meanwhile, strains C2L11 and C2L12B were isolated from the second instar larvae of the same host species; all three 16S rRNA gene sequences indicated that these three strains belong to the genus *Pseudomonas*, but they showed relatively low similarity to the closest type strains *P. congelans* DSM 14939T (97.4), *P. meliae* Ogimi 2^T^ (97.2), and *P. silesiensis* A3T (97.2), which suggested that they could belong to a new species within the genus. The phylogenetic analysis of both 16S rRNA and the MLSA of the housekeeping *gyrB, rpoB,* and *rpoD* gene sequences showed that the three strains locate together in a separated branch from those of the closest *Pseudomonas* type strains. ANIb and GGDC values confirmed that all three strains belong to the same species but are distant to the closest type strains, which confirm that they could be considered a new species [26,28,58,59]. Attending to phenotypical traits, it was observed that unlike the closest strains *P. rhizosphaerae* IH5^T^*, P. alkylphenolica* KL28^T^*, P. entomophila* L48^T^, and *P. congelans* P 538/23^T^, all three strains were able to weakly ferment glucose and to degrade urea. In addition, the three new isolates differ from *P. rhizosphaerae* IH5^T^ in the oxidase production and adipate assimilation tests, which are positive for the strains of this study, but negative for *P. rizhosphaerae* IH5^T^. Moreover, unlike *P. alkylphenolica* KL28^T^, *P. entomophila* L48^T^, and *P. congelans* P 538/23^T^*,* which are positive for gelatin hydrolysis, N-acetyl-glucosamine and the phenylacetate assimilation test, strains CA3A^T^, C2L11, and C2L12B showed negative results. Thus, all these results support the classification of strains CA3A^T^, C2L11, and C2L12B as a new species within the genus *Pseudomonas,* for which the name *P. typography* is proposed.

Apart from that, and in order to evaluate the potential role of these strains on their bark beetle host, an in silico assessment of the genetic potential of the three genome sequences was performed.

Adult bark beetles perforate the external bark to reach the inner bark, where their life cycle will be developed. Firstly, galleries are excavated along the tree and eggs will be laid. Once larvae are born, they excavate their own galleries, develop, and proceed through all the life stages (pupae, young adult, and adults). Once the adult stage is achieved, individuals will leave the tree and seek for a new host to start the cycle again. Thus, almost all of their life cycle develops inside the inner bark, which is composed mainly of cellulose, hemicellulose, and lignin [1,3,4]. Bark beetle cannot digest these compounds by themselves, non-profiting a potential carbon source. It has been proposed that the bark beetle bacteriome might hydrolyze these compounds into simpler sugars, which is potentially profitable for the beetle [1,4,54]. In silico analysis of the genome sequences predicted the presence of CDs related to several CAZYmes families that could be related with this activity, such as GH 3, GH 10, GH 13, GH 15, GH 39, and GH 94, CE 4, AA 3, and PL 4 and PL 26 families. During the hydrolyzation of hemicelluloses (xylans, mannans, mixed linkage β-glucans, and xyloglucans) and cellulose to glucose, the last step will be done by β-glucosidases, which are important enzymes during this process [60]. In *P. typographi* strains, genomes GH 3 and GH 39 were predicted. These families include this enzyme, among other CAZYmes that could be involved in the process, such as β-xylosidases (EC 3.2.1.21), α-l-arabinofuranosidase (EC 3.2.1.55), exoglucanase (EC 3.2.1.-), and exo-xyloglucanase (EC 3.2.1.155) [61]. In addition, the CE 4 family includes acetyl xylan esterase (EC 3.1.1.72) [61]. All these enzymes participate in cellulose and hemicellulose breakdown. The GH 94 and AA 3 families include cellobiose-related enzymes that can also participate in the hydrolysis process [60]. The GH 10 and 13 families include α-amylase (EC 3.2.1.1) and α-glucosidase (EC 3.2.1.20), the GH 15 family includes glucoamylase (EC 3.2.1.3), and PL 4 and 26 include rhamnogalacturonan endolyase (EC 4.2.2.23) and rhamnogalacturonan exolyase (EC 4.2.2.24), respectively. All these enzymes are related to starch and pectin hydrolysis, which are part of *Picea* bark and wood composition [34,35,62,63]. In order to confirm these enzymatic activities, in vitro assays were performed; pNPs assay confirmed the synthesis of α-glucosidase, β-glucosidase, α-xylosidase, β-xylosidase, and cellobiohydrolase enzymes, and the presence of halos in the plates with CMC, xylan, starch, and pectin as substrates confirmed the hydrolysis capacity of *P. typographi* stains. This trait has already been proposed and described for bark beetle bacteriome, including *Pseudomonas* strains [1,4,15,54].

Furthermore, tree’s phloem is known as a sugar-rich source but it is poor in phosphorus, nitrogen, and vitamins, among other important biomolecules and nutrients for the beetle development, for which an important role that could be played by the bacteriome is providing the necessary biomolecules for the beetle, turning non-assimilable mineral compounds to bioassimilable nutrients for the beetle, and more yet to describe [1,2,7,8,13,15]. In this regard, in silico analysis of the genome predicted the potential capacities of these three strains related to metabolic pathways for the reduction of adenyl sulfate (APS) to bioassimilable sulfide, which could suppose a sulfur source for the beetle. In addition, genes related to proteins involved in the biosynthesis of thiamine (vit. B1), riboflavin (vit. B2), pyridoxine (vit. B6), nicotinate (vit. B3), biotine (vit. B8), and folate (vit. B9) were annotated. These are very important for the beetles, since vitamin B is essential for them and they cannot synthetize [8].

Regarding protection against pathogens, this may occur through the inhibition of pathogens of the beetle itself or to other beetle’s symbionts [1,13]. Genome sequences analyses showed that these strains might have antimicrobial potential. In all three genomes, genes belonging to GH 3, GH 19, and GH 23 families, in which chitinase (EC 3.2.1.14) and exo-1,3/1,4-β-Glucanase (EC 3.2.1.-) CAZymes are included, were predicted. Chitinases and glucanases are very important enzymes in fungal cell wall hydrolysis and growth inhibition, which have already been described in *Pseudomonas* strains [64,65]. In addition, BGCs related to a hypothetical defense role were predicted, such as a biosynthetic bacteriocine cluster and an NRPS-like cluster, similar to AMB from *P. aeruginosa* PAO1 (40% similarity) [66]. Additionally, a BGC similar to crochelin A that could be related to siderophore activity was predicted [67]. AMB has already been proven as a relatively effective virulence factor against protozoa but only at relatively high concentrations [68]. Siderophore molecules can inhibit fungal growth, as e.g., *P. aeruginosa* siderophores have already been tested as effective against *Fusarium* and *Aspergillus* strains [69]. To confirm these predictions, in vitro siderophore assay was performed, and positive results for the three strains were obtained. Moreover, antibiosis tests using several filamentous fungal entomopathogenic strains of *Ips* beetles were accomplished; these tests confirmed the capability of the three strains to inhibit the growth of these fungal entomopathogens. Antimicrobial activity has already been described for other bark beetle isolates, e.g., *P. bohemica* [13]. Thus, the results obtained in this study support the hypothesis of a potential role of *Pseudomonas* associated to bark beetles in the protection of their host. In vivo tests analyzing the beetle survival improvement with the addition of these bacteria to reared beetles should be performed in the future in order to confirm this hypothesis.

In regard to the detoxification of environmental toxic compounds, such as the aromatics emitted by the tree when it is under attack, the analyses of the genome sequences of all three strains predicted routes related to p-hydroxybenzoic acid (PHBA) and salicylate into catechol degradation. Both are important aromatic compounds synthetized by the tree as chemical defenses against pathogens [70]. Although in vitro and in vivo tests should be performed to confirm the capability of these strains to degrade these aromatics, this role has already been described for *Dendroctonus ponderosae* bacteriome [71].

A NAGGN cluster had potential activity as osmotic stress resistance, as described for *P. aeruginosa* [72]. Finally, a carotenoid BGC was also predicted; generally, these pigments cannot be synthesized by animals, and it has already been described that some insect symbionts can synthesize them [73,74].

## 5. Conclusions

In this study, three closely related strains belonging to the genus *Pseudomonas* were isolated from individuals at different stages of the *I. typographus* life cycle. Genomes sequences of these three strains show their potential role in nutrient provisioning, protection against pathogens, and toxic compounds degradation. In vitro tests confirmed the capability of strains CA3A^T^, C2L11, and C2L12B to inhibit fungal entomopathogens of the beetle, as well as their ability to hydrolyze cellulose, xylan, starch, and pectin. A polyphasic taxonomic approach indicated that these strains belong to a new species within the genus *Pseudomonas*, for which the name *P. typographi* has been proposed.

Although further analyses are needed to confirm that the predicted capabilities occur within *I. typographus* holobiont and benefit the host, we can conclude that the new species *P. typographi* sp. nov. has genetic potential to aid in its host ecology, and the isolation of members of this new species from two different stages of the life cycle of the beetle also supports the possible relevance of this species for the bark beetle holobiont.

### Description of Pseudomonas typographi sp. nov.

*Pseudomonas typographi* (ty.po.gra.phi. M.L. adj. related to *Ips typographus*, the bark beetle where the type strain was isolated from). Cells are Gram-negative, rod shaped, with approximately 1 µm length and 0.5–0.6 µm width. On TSA medium, the optimal growth temperature is 28 °C, although good growth happens in the range of 4 to 37 °C; on TSB medium, it tolerates NaCl from 0 to 4, with an optimal growth with 1 NaCl; the pH growth range is 6 to 10, with an optimum at 8. Cells are catalase and oxidase positive. Results in the API 20 NE system are positive in the reduction of nitrates, D-glucose fermentation, arginine DiHydrolase, urease, assimilation of D-glucose, L-arabinose, D-mannose, D-mannitol, potassium gluconate, caprate, adipate, and trisodium citrate were also positive, whereas indole production, gelatin hydrolysis, β-galactosidase, N-acetyl-glucosamine, D-maltose, and phenylacetate were negative. In API 50CH, the type strain produced acid from glycerol, erythritol, **l**-arabinose, d-ribose, d-xylose, d-galactose, d-glucose, d-fructose, d-mannose, **l**-rhamnose, inositol, d-sorbitol, d-maltose, gentiobiose, d-turanose, d-fucose, and d-arabitol.

The type strain, CA3A^T^ (= CECT 30101^T^ = LMG 31781^T^), was isolated from a bark beetle from the species *Ips typographus* in Czech Republic. The DNA G + C of the type strain is 62.1 mol.

## Figures and Tables

**Table 1:** p-nitrophenyl (pNP) substrates prepared and enzymatic reaction tested.

pNP Substrate	Enzyme Tested
PNP-α-d-glucopyranoside	α-glucosidase
PNP-β-d-glucopyranoside	β-glucosidase
PNP-β-d-cellobioside	cellobiohydrolase
PNP-α-d-xylopyranoside	α-xylosidase
PNP-β-l-xylopyranoside	β-xylosidase

**Table 2:** ANIb, digital DNA–DNA hybridization (dDDH) (identities/HSP length) and G + C mol% difference, results of comparison of strains CA3A^T^, C2L11, and C2L12B within them and the closest type strains.

Genomes	*P. typographi* CA3A^T^ (JAAOCA000000000)			*P. typographi* C2L11 (JAAOCB000000000)			*P. typographi* C2L12B (JAAOCC000000000)		
	ANIb	dDDH	G + C Diff.	ANIb	dDDH	G + C Diff.	ANIb	dDDH	G + C Diff.
*P. typographi* CA3A^T^ (JAAOCA000000000)	-	-	-	99.6	97.7	0.12	99.7	97.7	0.16
*P. typographi* C2L11 (JAAOCB000000000	-	-	-	-	-	-	99.7	97.7	0.16
*P. alkylphenolica* KL28^T^ (NZ_P009048.1)	77.2	24.1	1.98	77.3	24.2	1.86	77.3	24.1	2.02
*P. congelans DSM* 14939^T^ (NZ_FNJH01000010.1)	75.7	23.0	3.28	75.7	23.1	3.16	75.7	23.0	3.32
*P. entomophila* L48^T^ (NC_008027.1)	77.6	24.6	1.56	77.5	24.6	1.67	77.6	24.5	1.51
*P. kribbensis* 46-2^T^ (NZ_CP029608.1)	76.5	23.5	2.06	76.5	23.5	1.94	76.5	23.5	2.10
*P. mandelii LMG* 21607^T^ (NZ_LT629796.1)	76.3	23.1	3.40	76.3	23.2	3.29	76.4	23.2	3.45
*P. meliae CFBP* 3225^T^ (NZ_JYHE01000001.1)	75.5	22.9	4.20	75.4	23.0	4.09	75.4	23.0	4.24
*P. rhizosphaerae* DSM 16299^T^ (NZ_CP009533.1)	77.2	24.8	0.61	77.2	24.9	0.49	77.2	24.8	0.65
*P. silesiensis* A3^T^ (NZ_CP014870.1)	76.6	23.7	3.02	76.5	23.6	2.91	76.5	23.6	3.06
*P. tremae* ICMP9151^T^ (NZ_LJRO01000117.1)	75.3	22.5	4.81	75.4	22.9	4.69	75.3	22.7	4.85

**Table 3:** Strain CA3A^T^, C2L11, and C2L12B genomes description based on RAST analysis.

Atribute	Strain (Value)		
	CA3A^T^	C2L11	C2L12B
Genome size (bp)	5,982,847	6,069,978	5,860,340
G + C content (%)	62.1	62.0	62.2
N50 value	141,379	127,151	133,313
L50 value	15	16	11
Number of contigs (with protein encoding genes)	229	158	174
Number of subsystems	364	362	364
Number of coding sequences	5965	5954	5775
Number of RNAs	63	60	64
Number of genes related to:			
Cofactors, vitamins, prosthetic groups, pigments	182	182	179
Cell wall and capsule	59	57	49
Virulence, disease, and defense	49	49	49
Potassium metabolism	7	7	7
Miscellaneous	44	44	44
Phages, prophages, transposable elements, plasmids	49	18	48
Membrane transport	122	107	103
Iron acquisition and metabolism	11	11	11
RNA metabolism	53	54	53
Nucleosides and nucleotides	102	101	102
Protein metabolism	201	198	200
Motility and chemotaxis	73	73	73
Regulation and cell signaling	36	37	36
Secondary metabolism	5	4	4
DNA metabolism	92	88	84
Fatty acids, lipids, and isoprenoids	126	119	120
Nitrogen metabolism	18	18	18
Respiration	123	124	121
Stress response	102	98	96
Metabolism of aromatic compounds	58	57	62
Amino acids and derivatives	463	458	455
Sulfur metabolism	87	85	79
Phosphorus metabolism	25	25	25
Carbohydrates	305	298	300

**Table 4:** dbCAN2 matches for strains CA3A^T^, C2L11, and C2L12B. EF * = Enzymatic Family, R * = Number of times the EF was predicted, and T * = No. of tools that predicted this EF.

EF *	R *	T *	CARBOHYDRATE-ACTIVE ENZYMES (CAZymes)
**AA**			
3	4	2	cellobiose dehydrogenase (EC 1.1.99.18); glucose 1-oxidase (EC 1.1.3.4); aryl alcohol oxidase (EC 1.1.3.7); alcohol oxidase (EC 1.1.3.13); pyranose oxidase (EC 1.1.3.10)
6	2	2	1,4-benzoquinone reductase (EC. 1.6.5.6)
12	1	2	pyrroloquinoline quinone-dependent oxidoreductase
**CBM**			
50	1	2	50 residues found attached to various enzymes from families GH18, GH19, GH23, GH24, GH25, and GH73
**CE**			
4	3	3	acetyl xylan esterase (EC 3.1.1.72); chitin deacetylase (EC 3.5.1.41); chitooligosaccharide deacetylase (EC 3.5.1.-); peptidoglycan GlcNAc deacetylase (EC 3.5.1.-); peptidoglycan N-acetylmuramic acid deacetylase (EC 3.5.1.-)
11	1	3	UDP-3-0-acyl N-acetylglucosamine deacetylase (EC 3.5.1.108).
**GH**			
0	1	2	Glycoside hydrolases not yet assigned to a family.
3	2	3	β-glucosidase (EC 3.2.1.21); xylan 1,4-β-xylosidase (EC 3.2.1.37); β-glucosylceramidase (EC 3.2.1.45); β-N-acetylhexosaminidase (EC 3.2.1.52); α-l-arabinofuranosidase (EC 3.2.1.55); glucan 1,3-β-glucosidase (EC 3.2.1.58); glucan 1,4-β-glucosidase (EC 3.2.1.74); coniferin β-glucosidase (EC 3.2.1.126); exo-1,3-1,4-glucanase (EC 3.2.1.-); β-N-acetylglucosaminide phosphorylases (EC 2.4.1.-); β-1,2-glucosidase (EC 3.2.1.-); β-1,3-glucosidase (EC 3.2.1.-); xyloglucan-specific exo-β-1,4-glucanase/exo-xyloglucanase (EC 3.2.1.155) and other.
10	1	3	α-amylase (EC 3.2.1.1); pullulanase (EC 3.2.1.41); cyclomaltodextrin glucanotransferase (EC 2.4.1.19); neopullulanase (EC 3.2.1.135); α-glucosidase (EC 3.2.1.20); maltotetraose-forming α-amylase (EC 3.2.1.60); isoamylase (EC 3.2.1.68); amylosucrase (EC 2.4.1.4); amylo-α-1,6-glucosidase (EC 3.2.1.33); α-1,4-glucan: phosphate α-maltosyltransferase (EC 2.4.99.16) and other.
13_11 + CBM48	2	3	α-amylase (EC 3.2.1.1); pullulanase (EC 3.2.1.41); cyclomaltodextrin glucanotransferase (EC 2.4.1.19); cyclomaltodextrinase (EC 3.2.1.54); trehalose-6-phosphate hydrolase (EC 3.2.1.93); oligo-α-glucosidase (EC 3.2.1.10); maltogenic amylase (EC 3.2.1.133); neopullulanase (EC 3.2.1.135); α-glucosidase (EC 3.2.1.20); isoamylase (EC 3.2.1.68); maltotriose-forming α-amylase (EC 3.2.1.116); branching enzyme (EC 2.4.1.18); trehalose synthase (EC 5.4.99.16); 4-α-glucanotransferase (EC 2.4.1.25); maltopentaose-forming α-amylase (EC 3.2.1.-); amylosucrase (EC 2.4.1.4); amylo-α-1,6-glucosidase (EC 3.2.1.33) and other.
13_16	1	3	α-amylase (EC 3.2.1.1); pullulanase (EC 3.2.1.41); cyclomaltodextrin glucanotransferase (EC 2.4.1.19); cyclomaltodextrinase (EC 3.2.1.54); trehalose-6-phosphate hydrolase (EC 3.2.1.93); oligo-α-glucosidase (EC 3.2.1.10); maltogenic amylase (EC 3.2.1.133); neopullulanase (EC 3.2.1.135); α-glucosidase (EC 3.2.1.20); maltotetraose-forming α-amylase (EC 3.2.1.60); isoamylase (EC 3.2.1.68); glucodextranase (EC 3.2.1.70); maltohexaose-forming α-amylase (EC 3.2.1.98); maltotriose-forming α-amylase (EC 3.2.1.116); branching enzyme (EC 2.4.1.18); trehalose synthase (EC 5.4.99.16); 4-α-glucanotransferase (EC 2.4.1.25); maltopentaose-forming α-amylase (EC 3.2.1); amylo-α-1,6-glucosidase (EC 3.2.1.33) and other
13_26	1	3	α-amylase (EC 3.2.1.1); pullulanase (EC 3.2.1.41); cyclomaltodextrin glucanotransferase (EC 2.4.1.19); cyclomaltodextrinase (EC 3.2.1.54); trehalose-6-phosphate hydrolase (EC 3.2.1.93); oligo-α-glucosidase (EC 3.2.1.10); maltogenic amylase (EC 3.2.1.133); neopullulanase (EC 3.2.1.135); α-glucosidase (EC 3.2.1.20); maltotetraose-forming α-amylase (EC 3.2.1.60); isoamylase (EC 3.2.1.68); maltohexaose-forming α-amylase (EC 3.2.1.98); maltotriose-forming α-amylase (EC 3.2.1.116); branching enzyme (EC 2.4.1.18); amylosucrase (EC 2.4.1.4); amylo-α-1,6-glucosidase (EC 3.2.1.33); and other.
13_3	1	3	α-amylase (EC 3.2.1.1); pullulanase (EC 3.2.1.41); cyclomaltodextrin glucanotransferase (EC 2.4.1.19); cyclomaltodextrinase (EC 3.2.1.54); trehalose-6-phosphate hydrolase (EC 3.2.1.93); oligo-α-glucosidase (EC 3.2.1.10); maltogenic amylase (EC 3.2.1.133); neopullulanase (EC 3.2.1.135); α-glucosidase (EC 3.2.1.20); maltotetraose-forming α-amylase (EC 3.2.1.60); isoamylase (EC 3.2.1.68); glucodextranase (EC 3.2.1.70); maltohexaose-forming α-amylase (EC 3.2.1.98); maltotriose-forming α-amylase (EC 3.2.1.116); maltopentaose-forming α-amylase (EC 3.2.1.-); amylosucrase (EC 2.4.1.4); malto-oligosyltrehalose synthase (EC 5.4.99.15); amylo-α-1,6-glucosidase (EC 3.2.1.33) and other
13_33	1	2	α-amylase (EC 3.2.1.1); pullulanase (EC 3.2.1.41); cyclomaltodextrin glucanotransferase (EC 2.4.1.19); cyclomaltodextrinase (EC 3.2.1.54); trehalose-6-phosphate hydrolase (EC 3.2.1.93); oligo-α-glucosidase (EC 3.2.1.10); maltogenic amylase (EC 3.2.1.133); neopullulanase (EC 3.2.1.135); α-glucosidase (EC 3.2.1.20); maltotetraose-forming α-amylase (EC 3.2.1.60); isoamylase (EC 3.2.1.68); glucodextranase (EC 3.2.1.70); maltohexaose-forming α-amylase (EC 3.2.1.98); maltotriose-forming α-amylase (EC 3.2.1.116); branching enzyme (EC 2.4.1.18); trehalose synthase (EC 5.4.99.16); maltopentaose-forming α-amylase (EC 3.2.1.-); amylosucrase (EC 2.4.1.4); amylo-α-1,6-glucosidase (EC 3.2.1.33) and other.
15	2	3	glucoamylase (EC 3.2.1.3); glucodextranase (EC 3.2.1.70); α,α-trehalase (EC 3.2.1.28); dextran dextrinase (EC 2.4.1.2)
19	2	2	chitinase (EC 3.2.1.14); lysozyme (EC 3.2.1.17)
23	2		lysozyme type G (EC 3.2.1.17); peptidoglycan lyase (EC 4.2.2.n1); chitinase (EC 3.2.1.14)
23 + CBM50	4	3	lysozyme type G (EC 3.2.1.17); peptidoglycan lyase (EC 4.2.2.n1); chitinase (EC 3.2.1.14)
24	CA3A^T^	2	lysozyme (EC 3.2.1.17)
39	1	3	α-l-iduronidase (EC 3.2.1.76); β-xylosidase (EC 3.2.1.37); α-l-arabinofuranosidase (EC 3.2.1.55); β-glucosidase (EC 3.2.1.21); β-galactosidase (EC 3.2.1.23)
65	1	3	α,α-trehalase (EC 3.2.1.28); maltose phosphorylase (EC 2.4.1.8); trehalose phosphorylase (EC 2.4.1.64); kojibiose phosphorylase (EC 2.4.1.230); trehalose-6-phosphate phosphorylase (EC 2.4.1.216); nigerose phosphorylase (EC 2.4.1.279); 3-O-α-glucopyranosyl-l-rhamnose phosphorylase (EC 2.4.1.282); 2-O-α-glucopyranosylglycerol: phosphate β-glucosyltransferase (EC 2.4.1.-); α-glucosyl-1,2-β-galactosyl-L-hydroxylysine α-glucosidase (EC 3.2.1.107)
73	1	3	lysozyme (EC 3.2.1.17); mannosyl-glycoprotein endo-β-N-acetylglucosaminidase (EC 3.2.1.96); peptidoglycan hydrolase with endo-β-N-acetylglucosaminidase specificity (EC 3.2.1.-)
77	1	3	amylomaltase or 4-α-glucanotransferase (EC 2.4.1.25)
94 + GT84	1	3	cellobiose phosphorylase (EC 2.4.1.20); laminaribiose phosphorylase (EC 2.4.1.31); cellodextrin phosphorylase (EC 2.4.1.49); chitobiose phosphorylase (EC 2.4.1.-); cyclic β-1,2-glucan synthase (EC 2.4.1.-); cellobionic acid phosphorylase (EC 2.4.1.321); β-1,2-oligoglucan phosphorylase (EC 2.4.1.-)
102	1	3	peptidoglycan lytic transglycosylase (EC 3.2.1.-)
103	2	3	peptidoglycan lytic transglycosylase (EC 3.2.1.-)
129	1	3	α-N-acetylgalactosaminidase (EC 3.2.1.49);
**GT**			
0	1	2	Glycosyltransferases not yet assigned to a family
1	1	3	UDP-glucuronosyltransferase (EC 2.4.1.17); zeatin O-β-xylosyltransferase (EC 2.4.2.40); 2-hydroxyacylsphingosine 1-β-galactosyltransferase (EC 2.4.1.45); N-acylsphingosine galactosyltransferase (EC 2.4.1.47); flavonol 3-O-glucosyltransferase (EC 2.4.1.91); anthocyanidin 3-O-glucosyltransferase (EC 2.4.1.115); sinapate 1-glucosyltransferase (EC 2.4.1.120); indole-3-acetate β-glucosyltransferase (EC 2.4.1.121); flavonol l-rhamnosyltransferase (EC 2.4.1.159) and other
2	5	3	cellulose synthase (EC 2.4.1.12); chitin synthase (EC 2.4.1.16); dolichyl-phosphate β-d-mannosyltransferase (EC 2.4.1.83); dolichyl-phosphate β-glucosyltransferase (EC 2.4.1.117); N-acetylglucosaminyltransferase (EC 2.4.1.-); N-acetylgalactosaminyltransferase (EC 2.4.1.-); hyaluronan synthase (EC 2.4.1.212); chitin oligosaccharide synthase (EC 2.4.1.-); β-1,3-glucan synthase (EC 2.4.1.34) and other.
4	6	3	sucrose synthase (EC 2.4.1.13); sucrose-phosphate synthase (EC 2.4.1.14); α-glucosyltransferase (EC 2.4.1.52); lipopolysaccharide N-acetylglucosaminyltransferase (EC 2.4.1.56); phosphatidylinositol α-mannosyltransferase (EC 2.4.1.57); GDP-Man: Man1GlcNAc2-PP-dolichol α-1,3-mannosyltransferase (EC 2.4.1.132) and other.
5	1	3	UDP-Glc: glycogen glucosyltransferase (EC 2.4.1.11); ADP-Glc: starch glucosyltransferase (EC 2.4.1.21); NDP-Glc: starch glucosyltransferase (EC 2.4.1.242); UDP-Glc: α-1,3-glucan synthase (EC 2.4.1.183) UDP-Glc: α-1,4-glucan synthase (EC 2.4.1.-)
9	2	3	lipopolysaccharide N-acetylglucosaminyltransferase (EC 2.4.1.56); heptosyltransferase (EC 2.4.-.-).
19	1	3	lipid-A-disaccharide synthase (EC 2.4.1.182).
28	1	3	1,2-diacylglycerol 3-β-galactosyltransferase (EC 2.4.1.46); 1,2-diacylglycerol 3-β-glucosyltransferase (EC 2.4.1.157); UDP-GlcNAc: Und-PP-MurAc-pentapeptide β-N-acetylglucosaminyltransferase (EC 2.4.1.227); digalactosyldiacylglycerol synthase (EC 2.4.1.241)
30	1	3	CMP-β-KDO: α-3-deoxy-d-manno-octulosonic-acid (KDO) transferase (EC 2.4.99.-).
35	1	3	glycogen or starch phosphorylase (EC 2.4.1.1).
51	3	3	murein polymerase (EC 2.4.1.129).
104	1	2	dTDP-β-l-Rhap: arginine α-l-rhamnosyltransferase (EC 2.4.1.-)
**PL**			
4	1	2	rhamnogalacturonan endolyase (EC 4.2.2.23).
5_1	1	3	alginate lyase (EC 4.2.2.3); endo-β-1,4-glucuronan lyase (EC 4.2.2.14)
7_1	4	2	poly(β-mannuronate) lyase/M-specific alginate lyase (EC 4.2.2.3); α-L-guluronate lyase/G-specific alginate lyase (EC 4.2.2.11); poly-(MG)-lyase/MG-specific alginate lyase (EC 4.2.2.-); endo-β-1,4-glucuronan lyase (EC 4.2.2.14); oligoalginate lyase/exo-alginate lyase (EC 4.2.2.26)
26	1	2	rhamnogalacturonan exolyase (EC 4.2.2.24).

* Annotations for explanation of abbreviates.

**Table 5:** Antagonism test of *P. typographi* strains against the entomopathogen fungi: *Lecanicillium muscarium* CCF3297 (1), *Beauveria brongniartii* CCF1547 (2), *Metarhizium anisopliae* CCF0966 (3), *Beauveria bassiana* CCF5554 (4), *Lecanicillium muscarium* CCF6041 (5), *Isaria farinosa* CCF4808 (6), *Beauveria bassiana* CCF4422 (7), and *Isaria fumosorosea* CCF4401 (8).

Strain	1	2	3	4	5	6	7	8
CA3A^T^	T	T	T	PI	PI	PI	T	T
C2L11	T	T	T	T	T	T	T	T
C2L12B	PI	PI	T	T	T	T	PI	PI

T = total inhibition. PI = partial inhibition.

**Table 6:** Phenotypic differences between *P. typographi* CA3A^T^ (1), C2L11 (2), and C2L12B (3) (data from this study) and their closest related species *P. rhizosphaerae* IH5^T^ [55] (4), *P. alkylphenolica* KL28^T^ [56] (5), *P. entomophila* L48^T^ [51] (6), and *P. congelans* P 538/23^T^ [57] (7).

TEST	1	2	3	4	5	6	7
Oxidase	+	+	+		+	+	-
**Growth:**							
>37 °C	+	+	+	+	ND	+	ND
>7% NaCl	+	+	+	ND	+	-	ND
**API 20NE**							
Reduction of nitrates	W	W	+	+	+	-	-
Glucose fermentation	W	W	W	-	ND	-	-
Arginine dihydrolase	+	+	+	-	ND	+	-
Urease	+	+	+	-	ND	-	-
Esculin hydrolysis	-	-	-	-	ND	-	+
Gelatin hydrolysis	-	-	-	-	+	+	+
Assimilation of:							
l-Arabinose	+	+	+	+	+	-	+
d-Mannose	W	W	W	+	+	+	+
N-Acetyl-glucosamine	-	-	-	-	+	+	-
Adipate	W	W	W	-	ND	-	ND
Malate	W	W	W	+	ND	+	+
Trisodium citrate	+	W	W	+	ND	+	ND
Phenylacetate	-	-	-	-	+	+	ND
**API 50CH**							
Glycerol	W	+	-	+	ND	ND	+
Erythritol	+	-	-	+	ND	-	+
d-Arabinose	-	-	-	+	ND	ND	ND
d-Adonitol	-	-	-	+	ND	-	-
d-Galactose	+	+	+	+	ND	-	+
l-Sorbose	-	-	-	+	ND	ND	ND
l-Rhamnose	+	W	W	+	ND	-	-
Dulcitol	-	-	-	+	ND	ND	ND
Inositol	W	-	-	+	ND	ND	+
d-Sorbitol	+	-	-	+	ND	-	+
d-Melibiose	-	W	W	ND	-	-	-
d-Saccharose (sucrose)	-	-	-	ND	ND	-	+
d-Trehalose	-	-	-	ND	ND	-	+
Glycogen	-	-	-	ND	W	W	-
Gentiobiose	W	+	+	+	ND	-	-
d-Turanose	+	-	-	+	ND	-	-
d-Tagatose	-	-	-	+	ND	ND	ND
l-Fucose	-	-	-	+	ND	W	-

+, positive; -, negative; W, weak; ND, not determined.
